# The Roles of IL-17, IL-21, and IL-23 in the *Helicobacter pylori* Infection and Gastrointestinal Inflammation: A Review

**DOI:** 10.3390/toxins13050315

**Published:** 2021-04-28

**Authors:** Astri Dewayani, Kartika Afrida Fauzia, Ricky Indra Alfaray, Langgeng Agung Waskito, Dalla Doohan, Yudith Annisa Ayu Rezkitha, Abdurachman Abdurachman, Takashi Kobayashi, Reny I’tishom, Yoshio Yamaoka, Muhammad Miftahussurur

**Affiliations:** 1 Department of Infectious Disease Control, Faculty of Medicine, Oita University, Yufu 879-5593, Japan; astri.dewayani@fk.unair.ac.id (A.D.); takashik@oita-u.ac.jp (T.K.); 2 Department of Anatomy, Histology and Pharmacology, Universitas Airlangga, Surabaya 60131, Indonesia; abdurachman@fk.unair.ac.id; 3 Institute of Tropical Disease, Universitas Airlangga, Surabaya 60115, Indonesia; kartikafauzia@gmail.com (K.A.F.); rickyindraalfaray@gmail.com (R.I.A.); langgengaw@gmail.com (L.A.W.); doctordoohan@gmail.com (D.D.); 4 Department of Environmental and Preventive Medicine, Faculty of Medicine, Oita University, Yufu 879-5593, Japan; yyamaoka@oita-u.ac.jp; 5 Department of Public Health and Preventive Medicine, Faculty of Medicine, Universitas Airlangga, Surabaya 60131, Indonesia; 6 Faculty of Medicine, University of Muhammadiyah Surabaya, Surabaya 60113, Indonesia; yudithannisaayu@gmail.com; 7 Department of Medical Biology, Faculty of Medicine, Universitas Airlangga, Surabaya 60132, Indonesia; ritishom@fk.unair.ac.id; 8 Gastroentero-Hepatology Division, Department of Internal Medicine, Faculty of Medicine-Dr. Soetomo Teaching Hospital, Universitas Airlangga, Surabaya 60286, Indonesia; 9 Department of Medicine, Gastroenterology and Hepatology Section, Baylor College of Medicine, Houston, TX 77030, USA; 10 Global Oita Medical Advanced Research Center for Health (GO-MARCH), Yufu 879-5593, Japan

**Keywords:** CD4 T cell, *Helicobacter pylori*, gastrointestinal, cancer, T helper-17, adaptive immunity, inflammation

## Abstract

Although millions of people have been infected by *Helicobacter pylori* (*H. pylori*), only a small proportion of infected individuals will develop adverse outcomes, ranging from chronic gastritis to gastric cancer. Advanced development of the disease has been well-linked with chronic inflammation, which is significantly impacted by the adaptive and humoral immunity response. From the perspective of cellular immunity, this review aims to clarify the intricate axis between IL-17, IL-21, and IL-23 in *H. pylori*-related diseases and the pathogenesis of inflammatory gastrointestinal diseases. CD4^+^ helper T (Th)-17 cells, with the hallmark pleiotropic cytokine IL-17, can affect antimicrobial activity and the pathogenic immune response in the gut environment. These circumstances cannot be separated, as the existence of affiliated cytokines, including IL-21 and IL-23, help maintain Th17 and accommodate humoral immune cells. Comprehensive understanding of the dynamic interaction between molecular host responses in *H. pylori*-related diseases and the inflammation process may facilitate further development of immune-based therapy.

## 1. Introduction

*Helicobacter pylori* (*H. pylori*) infection has become a global concern since it affects more than half of the human population and has been proven to initiate a carcinogenesis pathway [1]. *H. pylori* has a high recombinant rate, making it one of the bacteria with high variety, especially in the virulence profile [2]. For example, *H. pylori* strains are more pathogenic in the presence of the cagA gene than in its absence [3], which might explain the diverse symptoms and disease associated with *H. pylori.* Although most patients are asymptomatic, approximately 10% develop peptic ulcers, 1–3% develop gastric cancer (GC), and 0.1% develop mucosa-associated lymphoid tissue (MALT) lymphoma [4]. The risk of obtaining diseases after *H. pylori* infection is also associated with both the geographical area and ethnicity; people in Asian countries have the highest prevalence of GC [5,6]. These findings indicate that the outcomes of infection rely on the host–pathogen interaction, inflammatory responses, host genetic diversity, and environmental factors.

The chronic inflammatory reaction to *H. pylori* infection damages the gastric mucosa. Interaction between T helper (Th) cells and antigen presenting cells (APCs) during the infection skews the adaptive immune cell into Th-polarizing-cytokines. Studies have reported that *H. pylori*-specific gastric mucosal T cell responses are usually Th1 predominant, but recently, Th17—markedly interleukin (IL)-17—is believed to be one of the driving immune cells in *H. pylori* infection [7,8]. The Murine model demonstrated that IL-17A and IL-17 F cytokines play a role in neutrophil recruitment for mucosal immunity to extracellular pathogens. Although it is advantageous for host defense [9], neutrophil activation and recruitment become the cellular point in *H. pylori* inflammation lesions [10].

IL-17 has many associated cytokines, such as IL-1β, IL-6, IL-21, and IL-23. Studies have affirmed that IL-17, IL-21, and IL-23 are related to autoimmune diseases, allergies, and pathogen immunity [9,11,12]. The IL-17/IL-21 and IL-17/IL-23 axes are involved in maintaining Th17 expansion and have antimicrobial and even multiple inflammatory and hematopoietic effects on epithelial, endothelial, and fibroblast cells [13,14]. Together, the cytokines axes intertwine in the gastritis process. IL-21 itself has pleiotropic action on adaptive and innate immune cells, increasing proinflammatory cytokines released from macrophages, enhancing proliferation of lymphoid cells, and promoting B cell differentiation, whereas the expression of IL-23 in patients with *H. pylori* infection is elevated and positively correlates with the degree of neutrophils and monocyte infiltration [15].

This review aims to elaborate on the interaction of host immunity with *H. pylori*, focusing on IL-17, IL-21, and IL-23. Understanding the relation between cytokines is necessary to better comprehend the pathogenesis of the *H. pylori* infection and gastrointestinal-related inflammation diseases.

## 2. Bacterial Antigen Induced Immune Response

In order to colonize in the extreme conditions of the human stomach, *H. pylori* produces a number of bacterial virulence factors that play an important role in immune invading mechanisms. The most well-studied bacterial components of *H. pylori* are cytokine-associated gene A (cagA) and vacuolating cytotoxin A (vacA). CagA is a part of the 40 kb gene cluster famously known as the cag pathogenicity island (cagPAI) that encodes the Cag type IV secretion system (CagT4SS), which contributes to the pathogenicity of *H. pylori* [16]. The CagT4SS delivers *H. pylori* components into the host through the outer membrane protein (OMP) [17]. Evidence shows that the cagA product is involved in T cell activation and the induction of proinflammatory cytokines in the host cell, resulting in more severe gastritis and higher prevalence of peptic ulcer or GC. Patients with the cagA-positive strain of *H. pylori* showed a Th1-mediated cellular response in early carcinogenesis, followed by Th2-mediated cell immunity in the advanced stage, but there was no such tendency in cagA-negative patients [18]. Injection of cagA into the gastric mucosal can also induce nuclear factor κB (NF-kB)-mediated IL-8 production [19], and *H. pylori* containing complete cagPAI induces significantly higher levels of IL-8 [20]. It is reported that IL-8 has a positive correlation with IL-17. In the study by Tanaka et al., IL-17C mRNA correlated with IL-8 mRNA in cagA-positive patients [21]. Another study demonstrated that CagA downregulated the B7-H2 ligand to inhibit Th17 differentiation and evade the early clearance mechanism [22]. Few reports have focused on CagA and IL-21 or IL-23, but studies have shown that IL-23 and IL-21 were comparable in *H. pylori* patients with or without cagA [7,23,24].

Besides T cells, the complex influence of *H. pylori* on the immune response via APCs is represented by macrophages, dendritic cells (DCs), and B cells. This effect has been observed in DC maturation and alteration of antigen presentation, while other studies have provided evidence of “tolerogenic” DCs [25,26,27]. The expression of DC-induced proinflammatory cytokines IL-6, IL-12, IL-1β, and IL-23 are more prevalent in vitro cultures with *H. pylori* [25,28]. Transcription factor E2F1 is a DC maturation regulator that is downregulated in DC maturation. *H. pylori vacA* sustains immature states of DC by retrieving E2F1. Moreover, *vacA* suppresses costimulatory factors (CD40, CD86, MHC-II) in DC that might dampen T cells and promoted tolerance [29]. Another protein, *H. pylori* neutrophil-activating protein (*H. pylori*-NAP), triggers IL-23 production from neutrophils and monocytes [13].

Urease protein in combination of subunit A (UreA) and subunit B (UreB) is another abundantly produced protein from *H. pylori*, comprising up to 5% of the bacterial cell protein. UreB contributes to colonization and induces strong immune responses. CD4^+^ T cells from *H. pylori*-infected mice were co-cultured with recombinant UreB in the presence of macrophage as the APC was potent enough to induce Th17 number as well as IL-17A levels. When given rUreB as an immunization agent, it could prompt UreB-specific Th17 cells and thereby reduce bacterial colonization [30]. This shows urease as an important protein to induce Th17 response both in vitro and in vivo. Although some studies indicate *H. pylori* virulence is related to IL-17, the correlation with IL-21 and IL-23 requires further elucidation.

## 3. Overview of IL-17, IL-21, and IL-23

### 3.1. IL17

IL-17 comprises a family of related cytokines (IL-17A-F) and is majorly produced by a specific subset of CD4^+^ T helper (Th) 17 cells. To a lesser extent, Th17 also produces TNF-α, IL-6, IL-21, IL-22, and granulocyte macrophage-colony stimulating factor (GM-CSF) [13]. The Th17 lineage develops independently from Th1 and Th2 differentiation.

**Figure 1:**
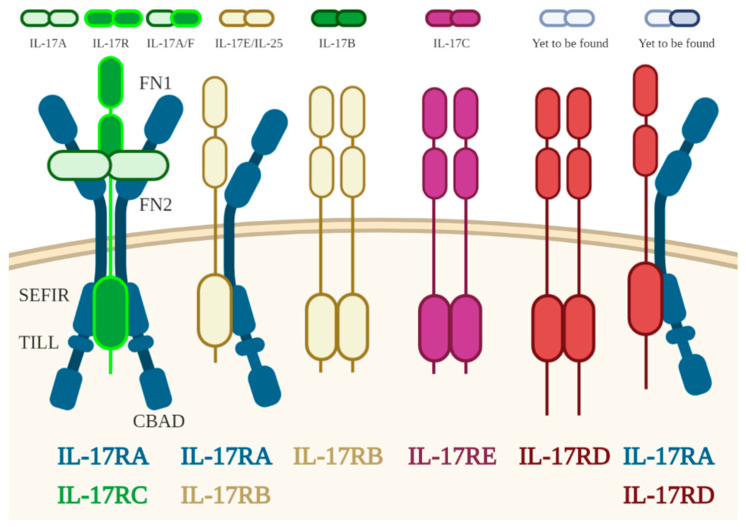
Illustration of IL-17 receptor family, receptor–ligands correlation, and their main structural appearance. FN, fibronectin III-like domain; SEFIR, SEF/IL-17R-related signaling domain; TILL, TIR-like loop; CBAD, C/EBPβ activation domain.

To date, five receptors have been identified as part of the IL-17 receptor family (Figure 1). This family consists of receptor subunits IL-17A, IL-17B, IL-17C, IL-17D, and IL-17E. Many of the genes encoding the IL-17R family are associated with clusters on mouse chromosome 6 and 14 and also on human chromosome 3. Despite the abundance of information regarding IL-17R and its ligand, the receptor for IL-17D is yet to be known, and the existence of ligands for IL-17RD/SEF or an IL-17RA/IL-17RD pairing have not yet been successfully proven [31,32]. Their correlation with the molecular developmental pathways of human Th17 cells might become clearer in future.

Although the molecular pathways regulating Th17-cells development in humans have not been fully explicated, studies in murine indicate the Th17 cell differentiation is driven by the transcription factor, retinoic acid-related orphan receptor gamma-t (RORγ), in the presence of IL-6 and TGF-β1 and then expanded by IL-23 [33,34]. There are similarities among members of Th17, but IL-17F has the highest amino acid homology with IL-17A and both cytokines might be secreted as the IL-17A/F heterodimer [10].

Similar to the innate immune receptors, such as IL-1R and toll-like receptors (TLRs), IL-17A activates NF-κB, a well-known transcription factor correlated with the inflammation process [35]. IL-17A can activate the p50 and p65 subunits of the classical NF-κB pathway. This activation leads to NF-κB-inducing kinase (NIK) mediation events in the non-classical pathway [36]. IL-17 is capable of activating NF-κB and c-Jun N-terminal kinase (JNK) in the presence of TNF receptor-associated factor 6 (TRAF6). Supporting this finding, several studies also concluded a critical role for TRAF6 as an adaptor for various signals, such as TNFRs as well as the IL-1 receptor—including IL-17R [37]. A previous study exhibited that in embryonic fibroblasts of TRAF6 deficient mice, IL-17 was incapable of activating the IkappaB kinases and JNK. This confirms that the lack of TRAF6 seems to be the crucial point for the failure to respond to IL-17, although transient transfection of TRAF6 expression plasmid into the TRAF6-deficient cells could reverse the effect [38].

Th17-associated cytokines have been described in a growing list of diverse autoimmune diseases, such as multiple sclerosis, Crohn’s disease, psoriasis, and arthritis. Mice with IL-17, IL-23p19, or IL-12/IL-23p40 deficiency are resistant to EAE, IBD, or CIA, while the absence of Th1 affiliation, IL-12, or IFN-γ signaling worsens the disease [39]. In brief, the function of each member of IL-17 is unique but still not well understood. IL-17A is believed to have function in autoimmune pathology, neutrophil recruitment, and extracellular pathogen immunity. IL-17B-IL-17D was hypothesized to function in proinflammatory activities. IL-17E can induce Th2 and suppress Th17. IL-17F and IL-17A/F, whose ligand has yet to be found, are believed to function in neutrophil recruitment and immunity to extracellular pathogen [32]. Overall, the most widely studied member of the IL-17 family was IL-17A. This might be because IL-17A plays a critical role in host defense against microbial infection and tissue inflammation.

### 3.2. IL-21

IL-21 is another cytokine produced by Th17, as well as by T follicular helper (Tfh) cells, NK, and Th1 cells [40]. It has close structural similarities to IL-15, IL-2, and IL-4, and mediates its function via a heterodimeric receptor composed of IL-21R and the common γ-chain [41]. IL-21 signaling leads to the activation of the JAK-STAT signal, or MAPK in some cells. IL-21 has a role in the activation, proliferation, and survival of both CD4^+^ T cells and B cells, in the functional activity of CD8 T cells and NK cells, suppresses Treg differentiation, and inhibits the function of DCs. Studies have shown that IL-21 correlates with chronic autoimmune diseases or EAE [42]. IL-21 can synergize with TGF-β1 to induce Murine Th17 independently of IL-6 [43] through a similar condition by upregulated IL23R and RORγt expression on naïve T cells and impeded the development of TGF-β-induced FOXP3^+^ Treg cells [44].

**Figure 2:**
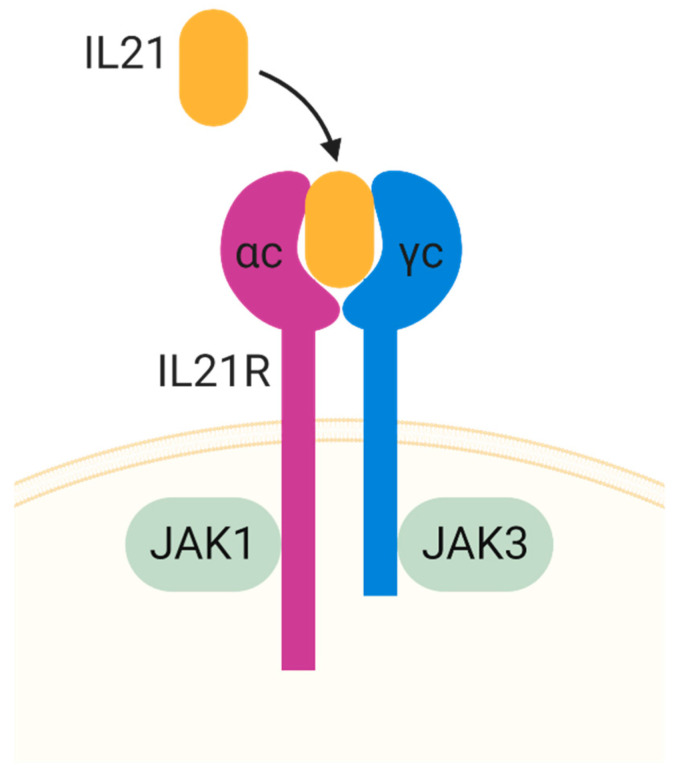
Illustration of IL-21 receptor and ligand. JAK, Janus Kinase.

IL-21R was discovered in 2000 as an orphan type I cytokine receptor. IL-21R is expressed in many cells, including immune cells (T cells, B cell, DC, and NK) and non-immune cells (epithelial cells, fibroblast, and endothelial cells). The similarity of IL-21 to IL-2, IL-4, and IL-15, suggests the possibility that IL-21 might share an important receptor on the common cytokine receptor γ chain (γc), encoded by IL2RG, the gene found to be mutated in humans with X-linked severe combined immunodeficiency (XSCID) [45]. It has similarities to the other γc family cytokines in activating Jak1 and Jak3, wherein IL-21R interacts with Jak1 and Jak3 interacts with γc (Figure 2) [46,47].

### 3.3. IL-23

**Figure 3:**
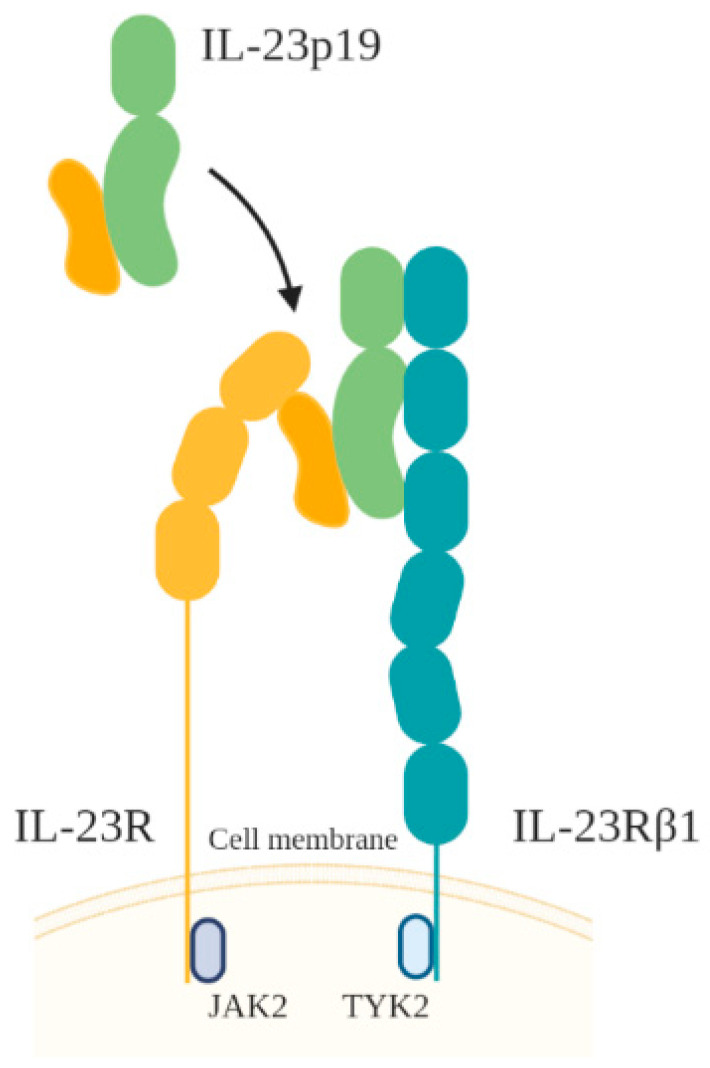
Illustration of IL-23 receptor and ligand.

IL-23, a heterodimer of the IL-23p19 subunit and an IL-12p40 subunit, is produced by APCs, including macrophages, DCs, and neutrophils. IL-23 has several homologies with IL-6 and G-CSF. The IL-23 receptor (IL-23R) itself is composed of IL-12R β1 combined with IL-23R, a specific receptor that resembles IL-6gp130 (Figure 3) [48,49]. The IL-23 signaling pathway is similar to that of IL-12, with a prominent role for STAT3 [48].

The function of IL-23 resembles that of IL-12 in linking innate responses and adaptive immunity. Both cytokines are increased in intestinal inflammation; however, IL-12 stimulates the IFNγ and Th1 lineage, whereas IL-23 does not directly stimulate IFNγ but rather acts on memory cells and enhances Th17. In vitro studies have shown that IL-23 does not drive naïve CD4^+^ T cell toward the Th17 phenotype; instead, later in Th17 development, the focus is on regulating IL-17 secretion through the STAT3-dependent pathway [13,39] or how collaboration with other pathways indirectly affects Th17 survival. In the absence of TGF-β, IL-23, in conjunction with other proinflammatory cytokines, IL-1β and IL-6, helps promote the differentiation of Th17 [50,51]. Another unique function of IL-23 is that it also acts as an end-stage effector cytokine through direct action on macrophages [52].

## 4. The Role of IL-17, IL-21, and IL-23 in Gastrointestinal Inflammation

An increase in the number of epithelial lymphocytes is not specified in *H. pylori* infection but could be found in other disorders, such as microbial infection, inflammatory bowel diseases (IBD), and celiac disease [9,39]. Th17-associated cytokines have well demonstrated both protective and pathogenic functions in the gut environment. However, IL-21 and IL-23 are not only linked with Th17 but are also cross-regulated with classical IFNγ-produced Th1.

In microbial colitis, the protective role of IL-17 is important for controlling *Citrobacter rodentium* (*C. rodentium*) oral infection [53]. Conversely, a pathogenic role is suggested by IL-23’s ability to stimulate IFNγ production by human memory T cells or in the *Helicobacter hepaticus* colitis model. The p19 subunit has been found to regulate mucosal rather than systemic immune responses in the colitis model [12]. IL-21, alongside IFNγ, IL-17, and IL-22, is induced in *C. rodentium*-infected mice for microbial clearance. Wild-type mice had a lower bacterial burden, yet their mucosal inflammation was more noticeable than in *Il21r* knockout mice. IL-21 is partially mediated downstream through STAT3, which is also important for IL-17. In this study, STAT3-deficient mice demonstrated a more impaired condition than *Il21r* knockout mice [54].

A previous meta-analysis reported possible crosstalk between *H. pylori* and IBD. A lower rate of *H. pylori* infection was found in IBD patients than that in non-IBD. This association remains controversial as several studies have reported that *H. pylori* infection protects against the progression of IBD [55,56], while a small number of studies have revealed no significant correlation [57]. IBD mainly consists of ulcerative colitis (UC) and Crohn’s disease (CD), which are multifactorial idiopathic diseases. A similar imbalance of Treg/Th17 in abnormal immune reaction likely contributes to the IBD process [55,58]. IBD patients project a higher expression of IL-17 and have a tendency toward a marked deficiency in Treg for controlling immune-mediated inflammation [59,60]. Comparably, Treg is induced in the *H. pylori* evading mechanism as well for limiting gastric inflammation, especially in children [61]. The infection elevates TGF-β but lowers the IL-17A, IL-17F, IL-6, IL-21, and IL-23 levels, thus likely affecting the immunosuppressive Treg balance across the colon environment in the colitis model [62].

Homodimeric IL-17A and IL-17F show a converse effect. The dextran sulfate sodium (DSS)-induced colitis mouse model showed exacerbated inflammation after IL-17A neutralization. IL-17A and IL-22 is believed to help strengthen tight-junction formation by inducing the expression of claudins in intestinal epithelial cells, stimulating mucin production, and enhancing goblet cell restitution [14]. The synergizing function also activates the expression of S100A8 and S100A9 (a component of calprotein, lipocalin, and the β-defensin protein) in epithelial cells [63], whereas IL-17F is suggested to exacerbate the inflammation DSS model. Mice deficient in IL-17F develop milder symptoms and present a lower mRNA expression, comparable to wild-type controls [14].

Th17 cytokines, as well as the IL-23 level, have seen an increase in the intestinal mucosa, plasma, and serum of IBD patients. Moreover, polymorphism in IL-23 or IL-17 pathways has been associated with an increasing risk of IBD [64,65]. From a molecular viewpoint, one study shows the early protective function of IL-17A in the gut mucosal barrier, which is produced by IL-23R^+^ RORγt^+^ T cells but IL-23 independent. IL-17A regulates occludin protein cellular localization and Act-1 activation during DSS injury, whereas IL-23 requires RORγt for transactivation to promote IL-17F. Neutralizing IL-23 but not IL-17A minimizes tissue inflammation [66].

IL-23 favors the pathogenic Th17 phenotype compared to cells cultured under TGF-β and IL-6 due to IL-23’s inability to induce IL-10 production for immune suppression [67]. Using Rag^−/−^ mice colitis model treated with IL-23R^−/−^ CD45RB^high^ and CD4^+^ cells alone were able to elevate IL-10 production, while co-transfer with wild-type T cells abrogated the action, implicating the capability of IL-23-responsive T cells to reduce IL-23R^−/−^ T cells response and produce IL-10 in the colonic mucosa. The Rag^−/−^ recipients’ IL-23R^−/−^ donor T cells also had increased FOXP3^+^ regulatory T (Treg) cells, which are important for maintaining intestinal homeostasis [68]. These findings suggest that IL-23 may promote intestinal inflammation through the function of T cells and by restraining the mucosal-protective population.

The role of IL-21 in Th17 and Th1 is prominent in chronically inflamed areas. In CD patient tissue, IL-21 was produced mostly by CD4^+^ T cells co-expressing IFNγ, with a small amount from Th17 [69]. UC patients displayed excessive IL-21 in mucosal samples, leading the colonic epithelial cells to produce the macrophage inflammatory protein (MIP)-3a (also called CCL20), a chemokine upregulated in IBD patients or mice with chemically induced colitis. Molecular signaling from IL-21 activates ERK 1/2 and p38 mitogen-activated protein (MAP) kinase, whereas the blockade of those pathways significantly inhibited MIP-3a. Thus, IL-21-deficient mice were protected from colitis through the reduction of Th17 and Th1 as well as MIP-3a/CCR6 interactions displaying the attraction of pathogenic Th17 cells into inflamed tissues [69,70]. Besides sustaining immune cells responses, IL-21 is capable of enhancing the production of matrix metalloproteinases (MMPs). Elevated expression of MMP-1, MMP-2, MMP-3, and MMP-9 by fibroblasts, and MMP-2 and MMP-9 by gastric epithelial cells following IL-21 induction may contribute to extra-vascularization, inflammation, and remodeling of the extracellular matrix (ECM) [11].

## 5. Th17 Roles in *H. pylori* Infection

The function of Th17 cells remains contradictory although they tend to display a proinflammatory function in the context of *H. pylori* disease. IL-17 is required to facilitate bacterial clearance, though the levels produced may be insufficient for extensive clearance and may cause IL-17-mediated-diseases from inflammation [71]. The IL-17 family could induce numerous immune regulatory and proinflammatory factors related to local tissue, such as antimicrobial peptides (b-defensin and S100 protein), chemokines (CXCL1, CXCL-5, CCL-2, CCL-20), and MMPs. This increased production of chemokines can cause inflammation-associated neutrophil infiltration [63,72].

In early colonization, *H. pylori* modulates immature “tolerogenic” DCs that skew the Th17/Treg balance toward Treg. *H. pylori*-infected patients with gastritis display an inverse correlation between the Th17/Treg ratio and bacterial density, indicating Tregs may reduce inflammation and provide pathogen persistent [61,71]. The plasticity of Th17/Treg is demonstrated by the ability of Treg to turn into Th17 or vice versa, where co-expression of both Treg and Th17 (FOXP3 and RORγt) signature genes reside in the same cell. TGF-β, alongside IL-6, IL-21, and IL-23, inhibits FOXP3 expression in a dose-dependent manner [73].

**Figure 4:**
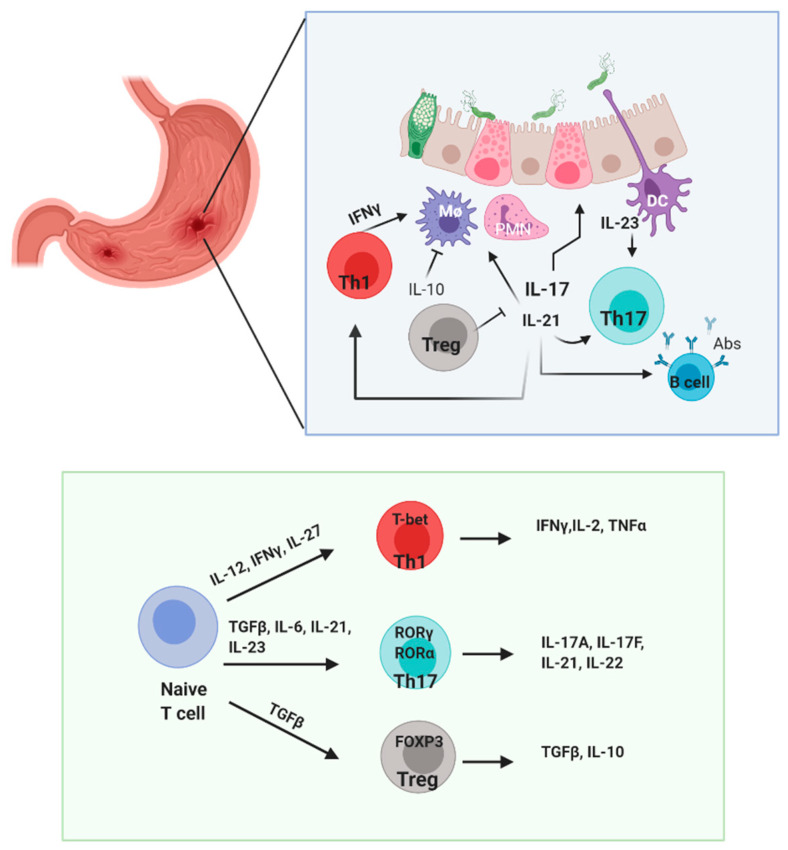
Illustration of immune cellular interaction in *H. pylori* infection. *H. pylori* is sensed by dendritic cell (DCs) and epithelial cells followed by the advent of CD4^+^ T cells, PMN, and macrophage to the gastric mucosa for bacterial clearance. However, continuous activation might increase local inflammation. Bacteria could modulate the escape mechanism and skew DCs to produce IL-23. Th17 equilibrium leans to the pathogenic side, along with Th1 releasing proinflammatory cytokines that recruit more PMNs. Regulatory T cells reportedly suppress the inflammatory effects of infection in the gastric mucosa. Treg, regulatory T cell; Th1, T helper produce IFNγ; Th17, T helper produce IL-17; DC, dendritic cell; Mϕ, macrophage; PMN, polymononuclear neutrophil; B cell, lymphocyte produce antibody.

Following that, the mouse model demonstrated that elevated IL-17, through the Th17 responses, precedes Th1 mediate mucosal inflammation. IL-17 production in the early response induces neutrophil activation for bacterial eradication but later contributes to chronic inflammation and thus favors pathogen infection [8,74]. In human cases, there is an elevated level of IL-17 and IFNγ in the gastric mucosa of *H. pylori* patients, and upregulation of Th17-related gene expression (IL-6, IL-23 p19, IL-12/IL-23 p40, TGF-β1) [7] has been found to progressively increase from gastritis to peptic ulcers [75]. The elevation of similar cytokine levels indicates the mixed responses of Th1/Th17 in the gastritis process (Figure 4).

IL-8 can trigger differentiation of Th17 cells through simultaneous activation of STAT3 with IL-6, while IL-17 is also able to activate ERK 1/2 MAP in the gastric epithelium to release IL-8, which increases the chemoattractant antigen to attract more neutrophils into the gastric mucosa [76]. This consequence of active synthesis of IL-8 might be involved in gastric ulcer development and tumorigenesis [77]. Increments of Th17 as well as IL-17 and IL-8 were positively correlated with *H. pylori* density in peptic ulcer disease but were negatively correlated in patients infected with gastritis [78]. Similar observations have been noted in IL-21 and IL-23 [15,23,24].

Synergistic combinations strongly modulate NF-κB; the important transcriptional factor participates in the immunoinflammatory response [79,80], releases oxidative stress (nitrite oxide, reactive oxygen stress), and therefore leverages mucosal damage and epithelial cell apoptosis [8,81]. The summarize cytokines can be seen in Table 1.

## 6. IL-17 and IL-21 Axis in *H. pylori* Infection

IL-21 has been found to be increased in patients with *H. pylori* infection and also in GC mucosa tissues [23,91]. To the proinflammatory side, molecular signaling shows STAT1 and STAT3 are important in Th17 and Th1. CD4^+^ T cells of IL21^−/−^ *H. pylori*-infected mice had lower phosphorylation of STAT1 and STAT3 than wild-type. Moreover, this led to lower expression of *tbx21* and *rorc* genes, which encode Tbet and RORγ. IFNγ and IL-17 can induce chemokine expression and neutrophils required to control the bacterial burden. Thus, mice with IL-21 deficiency had lower IFNγ and IL-17, and they were protected from chronic gastritis despite having more bacterial colonization [42]. IL-21 could also signal through epithelial cells to induce chemokine CCL20. Alongside *H. pylori* similar effect, the interaction of CCL20/CCR6 increases T cell subset mobilization [86]. On the other hand, being a pleiotropic cytokine, in vitro assay shows recombinant IL-21 has an immunomodulatory effect on *H. pylori*-cocultured DCs by reducing proinflammatory cytokines (IL-1β, IL-6, IL-12, and IL-23) [88]. In addition, IL-21 induction of MMP-2 and MMP-9 production in vivo via NF-κB activation might be related with chronic inflammation [23].

Other than Th17, IL-21 produced within the germinal center in lymph nodes or Peyer’s patches has demonstrated an autocrine loop for Tfh cell generation. In addition, B cells proliferate based on IL-21 responsiveness and might help to switch isotype or increase the antibody production [87]. In both humans and mice, *H. pylori* infection stimulates strong specific IgG and IgA antibodies in the blood and gastric mucosa. IL-17RA^−/−^ mice show an impaired negative feedback of Th17 from higher expressions of IL-17A and IL-21 in the stomach. At the chronic infection, these mice have a greater abundance of B cells infiltration and lymphoid follicles in the gut following enhancement of anti-*H. pylori* antibodies. This may imply that IL-21 drives B cells in the antibody response of IL-17RA^−/−^ mice [89]. In accordance, *H. pylori*-infected IL-21-deficient mice lack a *H. pylori*-specific antibody response in serum [42].

The importance of the antibody level is still in question. B cells in the gastric lumen have adapted to produce secretory IgA and IgM to facilitate bacterial clearance or neutralize toxins. Even so, because *H. pylori* uses several different receptors, one antibody ligand would not be effective for protection. Antibodies’ importance in the pathogenesis of gastritis could be on account of the capability of monoclonal antibodies against *H. pylori* to recognize an epitope on the gastric epithelium of mice and humans [92]. Mice injected with these antibodies experienced gastritis and mild erosions [93]. Interestingly, a study using B cell-deficient mice with *H. pylori*-specific antibody production had less inflammation but a prolonged eradication time [94]. However, the lack of B cells did not diminish gastritis.

These studies point to the possibility of a specific antibody biomarker to help predict patient outcome. In addition, while showing the impact on B cell proliferation, less is known about the IL-21 correlation with MALT lymphoma in gastric disease as it accounts for the parotid gland of Sjörgen patients [95].

## 7. IL-17 and IL-23 Axis in *H. pylori* Infection

The relationship between IL-17 and IL-23 tends toward the pathogenic side. The function of Th17 cells themselves differs between diseases depending on cytokine activation in the upstream pathway. It is reported that Th17 activated by TGFβ and IL-6 promotes barrier tissue integrity and mucosal defense and mitigates the pathogenic responses, while IL-23 drives Th17 cells to produce tissue inflammation in chronic infection, as well as granuloma formation and autoimmunity [90]. IL-23/IL-17 signaling increases neutrophil recruitment for extracellular bacterial clearance [82], but this can be conversely advantageous for bacterial invasion.

*H. pylori* colonization leads to the activation APCs, especially DCs, which have a central role in the induction of adaptive immune responses that produce cytokines involved in Th17 and Treg differentiation (Figure 4). Khamri et al. demonstrated that *H. pylori*-cultured DCs were able to induce IL-23 and significantly boosted IL-17 from CD4^+^ T cells compared to untreated DCs. They also showed that IL-23-positive DCs were present in human-infected samples [96].

IL-23 demonstrated a minor contribution to the development of chronic gastritis in a *H. pylori* infection mouse model 3–4 months post infection but not in acute inflammation. The IL-23^−/−^ mice had significantly decreased levels of IL-17 and IFNγ after chronic infection [97]. On the other hand, *H. pylori* co-culture with BMDCs can induce mRNA of IL-6, IL-1β, and IL-23p19, which might drive IL-17 expression via STAT3 [97]. The treatment of human lamina propia mononuclear cells with anti-IL-23 inhibited pSTAT3, which dampened IL-17 production [7]. There are some possible mechanisms by which STAT3 regulates IL-17. One possibility is that IL-23, through Jak2, activates STAT3 and NF-kB, both of which bind directly to the IL-17 promoter and thus enhance transcriptional activity [98]. Alternatively, STAT3 may enhance the IL-17 differentiation regulator, RORγt [99]. STAT3 could also enhance IL-23R expression, hence amplifying the feedback that maintains the IL-17/IL-23 pathway [7].

Another report from Koussoulas et al. showed that the mucosa of peptic ulcer and duodenal ulcer patients can potently secrete IL-23 related to the degree of neutrophil infiltration. They later demonstrated a significant elevation of IL-23 in patients with chronic gastritis but only minor elevation in those with peptic ulcers or duodenal ulcers after lipopolysaccharide (LPS) stimulation to demonstrate that apoptosis of gastric mucous cells could be enhanced by *H. pylori* LPS [15]. These studies indicate that IL-23 can either be an independent factor or a supporting mediator in gastric inflammation in response to *H. pylori* evading mechanisms.

## 8. IL-17, IL-21, and IL-23 Role in GC Development

The exact causes of GC remain poorly understood, but it has been widely reported that metastatic progression relies upon inflammation. The progression of GC is prominent in *H. pylori*-infected patients, and the frequency of cancer is significantly higher in patients with non-ulcer dyspepsia, gastric ulcers, and gastric polyps but not in those with duodenal ulcers [100].

Accumulating evidence indicates that the Th17/Treg proportion is increased in GC patients [101]. As previously described, Treg-favoring bacterial colonization and the inadequate clearance response from Th cells that later mediate inflammation and oxidative stress contribute to DNA damage [81]. Th17 responses in the pathogenesis of GC correlate with IL-17, IL-6, TGF-β, and IL-23 levels [102] and those corresponding with the severity [103]. The pro-tumorigenic of IL-17 also interrelates with IL-8, which is produced by many types of cancers [80]. Gastric myelofibroblast (GMF) as APC from both *H. pylori*-exposed and GC patients required elevated mRNA IL-6, TGF-β, and IL-21 to maintain Th17 induction [91]. This suggests that mucosal contact from chronic inflammation due to *H. pylori* infection contributed to Th17 production and GC development. Moreover, the Th17 immunity persists in serum even after bacterial eradication. The tenacity of IL-17A and IL-17F exposure is suggested to increase the risk of cancer [104].

Neutrophil recruitment by IL-17 in the tumor microenvironment can become a source of IL-17A and promote GC invasiveness [83]. In addition, IL-17 demonstrates the ability to produce pro-angiogenic factors, including VEGF, prostaglandin E1 (PGE1), PGE2, and macrophage inflammatory protein-2 (MIP-2), by fibroblasts as well as tumors, MMP-2, and MMP-9. Thus, IL-17 stimulation of vascular endothelial cell migration and cord formation can enhance angiogenesis and tumor growth [80,84,85].

As part of Th17, the level of IL-21 together with IL-17 is highly secreted from the T cells of gastric distal adenocarcinoma. Secreted peptidyl prolyl cis and trans-isomerase (HP0175) of *H. pylori* can drive those cytokines to contribute to matrix degradation, angiogenic pathways, and monocyte activation [105]. Ectopic IL-21R upregulation in epithelial GC patients could increase JAK/STAT pathways in gastric tumorigenicity [106].

Previous studies displayed the IL-17/IL-23 axis theory in inflammation-related malignancies, including gastric neoplasms. Cytokine levels were found to be positively correlated with stages. Furthermore, epidemiological analyses showed that Asian patients with selected polymorphisms in genes coding for both IL-17 and IL-23 might have an altered risk for developing GC [107,108]. Elucidation of possible mechanism of IL-23’s direct effect in GC tumorigenesis is still rare. Through in vitro experiment, Liu et al. showed co-cultured cell lines SGC-7901 and MKN45 with human recombinant IL-23A and *H. pylori* lysate induced IL-17A/IL-17RA/NF-κB activation [108], while Xu et al. demonstrated IL-23 promoting tumor cell migration via STAT3 in BGC-823 GC cell line [109].

## 9. Interleukin Polymorphism and the Development of Gastrointestinal Cancer

Varying levels of interleukin expression result in a different inflammatory effect, which influences the severity of diseases. Several host factors can influence the variation in expression, such as age, gender, the presence of autoimmune disease, and single nucleotide polymorphisms (SNPs) that present as a nucleotide variation between the allele [110]. SNPs could partially explain the different susceptibility to diseases among patients. IL-17, IL-21, and IL-23 polymorphism and their association in many diseases have been reported.

Several studies have reported that patients with a high expression of IL-17 had a better prognosis compared to those with a low expression, but some studies reported otherwise. Despite the controversy, SNPs are believed to code the variation of expression associated with GC. A meta-analysis evaluated the association of IL-17 rs1974226, rs2275913, rs3819024, rs4711998, and rs8193036 between 1126 GC patients and 1221 cancer free controls [111]. In this study, rs3819024 with the AA allele was associated with a decreased GC risk. The stratification study showed that IL-17 rs1974226 AA was associated with GC risk in the elderly (>59-years-old). The higher GC risk was independently associated with rs1974226 for AA compared to GG + GA. The GC risk was also significantly associated with the rs2275913 for GA + AA compared to AA. The variation of rs2275913 was located within IL-17A genes, also called IL-17A G197A polymorphism. The updated meta-analysis by Elshazli et al., including 7660 cases and 9409 controls, showed similar results for rs2275913 c-197G>A [112]. Another polymorphism in IL-17A was located in the rs3748067 c1249C>T and was also associated with GC risk as reported in the meta-analysis [112,113]. Polymorphism in IL-17F rs763780 was associated with the susceptibility of patients to GC. The association of IL17A rs2275913G>A and IL-17F rs763780C>T polymorphisms with other gastrointestinal cancers, such as hepatocellular carcinoma and colorectal cancer, were also evaluated. However, there was no statistically significant association, indicating a cancer-type-specific function [114]. They also found that the polymorphism was significantly associated with GC in those with an Asian ethnicity, but not in Caucasians [114]. These results indicate the strong role of the host factor to the susceptibility to GC.

Interleukin gene polymorphism was detected in IL-21 rs907715, rs2221903, and rs12508721. A recent study investigated the association of those SNPs with gastric precancerous lesions in 588 cases and 290 healthy controls, but only *IL-21* rs907715 CC + C was associated with atrophy and intestinal metaplasia [115]. Polymorphism can alter the regulation of transcription, protein translation, and cell proliferation.

To date, the SNPs of IL-23 genes and their association with GC have not been established. However, SNPs in the IL-23 receptor (IL-23R) were reported. In a study with 479 cases and 483 controls, patients with *IL-23R* rs1884444 GG and rs6682925 CC genotype had less survival. Moreover, survival was poor in elderly patients with *IL-23R* rs1884444 GG and female participants with rs6682925 CC [116]. This result showed a possible utility of interleukin gene polymorphism to stratify the risk of patients to GC and choose the treatment strategy in addition to *H. pylori* infection.

## 10. Expert Opinion

It is always quite fascinating to understand the immune response involved in each step of pathogenesis. Gastrointestinal diseases, both autoimmune and *H. pylori*-related infection, are similar in regard to inflammation. Other than Th1, another subset—namely Th17—arises to mediate that process. Correlations between IL-17 levels and the inflammation rate in *H. pylori* patients with gastritis and gastric ulcer have been proven, though the double-edge sword of Th17 and its associated cytokines is still controversial. Research concerning Th17 differentiation has been performed in both human and animal models. However, the requirement factors in human Th17 differentiation need to be carefully interpreted since most studies used peripheral blood, considering more antigen exposure in particular subjects; thus, naïve cells may not be present in the starting population as in isolated blood from the cord or spleen. Understanding proliferation and differentiation of human T cell subsets in naïve and committed cells will help grasp the intricacies of Th17 cell responses.

Whether stimulation in the microenvironment can change T cell polarization depends on the acute or chronic standpoint. There is a possibility of FOXP3+ Treg to become Th17 under microenvironmental influence, and this plasticity is also displayed on Th17/Treg in human patients, yet the exact underlying mechanism of interconversion in the co-expression of T cells is still poorly understood. *H. pylori* has infected humans for almost 60,000 years and evolved escaping strategies to elude the immune system during colonization. The enigma of the remodeled immune balance is demonstrated by the fact that a patient with ulcer has better *H. pylori* clearance than one with chronic gastritis. During colonization in the chronic gastritis state, an abundance of immunosuppressive Treg could not efficiently eradicate the infection with fewer symptoms. On the other hand, patients with gastric ulcers tend to be in a more proinflammatory state, and the increase in IL-23 and IL-21 helps Th17 induce severe pathological lesions; however, *H. pylori* can be handled better with antibiotic and proton-pump inhibitors [75,117,118]. Similarly, recent studies have reported a negative correlation between *H. pylori* and the incidence of IBD. Although the possible mechanism remains unclear, the infection could elevate the immunosuppressive effects of Treg that likely spreads across the extra-gastric system and shows a protective side.

Alongside environmental factors, genetic factors also play a role in the alteration of cytokines in carcinogenesis. Polymorphism in IL-17 exhibits a link with *H. pylori* infection, which predisposes patients to the development of GC development increasing activity and inflammation [119]. Moreover, cascade signaling, such as TLR, could also increase susceptibility [120]. A Chinese population study discussed the association between IL-21 polymorphism and the risk of precancerous lesions [115], but the limited number of studies on IL-21 cannot describe the bigger picture.

In the time ahead, the exact mechanism whereby the regulatory immune response becomes protective or pathogenic will continue to be elucidated. Th-17 and IL-23 pathway variants have been associated with the risk of intestinal inflammation conditions; thus, the use of a cytokine inhibitor in gastrointestinal diseases has been studied in clinical trials. Selective IL-23 and Th17 agents, such as briakinumab, secunikumab, and brazikumab, have been administered to IBD patients in a phase 2 trial, which may elicit a promisin yet controversial result [121,122]. More research is needed to obtain a clearer picture of the neutralization effect and possible correlation to *H. pylori* colonization.

The pathogenicity of IL-17A is capable of inducing apoptosis in parietal cells of mouse gastric mucosa, similar to that seen in chronic inflammation. Neutralizing IL-17A reduced the atrophy severity compared with that in untreated mice, although the atrophy did not reverse completely [123]. In the future, these cytokines might become adjuvant treatment for solid tumors, such as GC. However, further studies identifying the cytokines with a potent role in GC or even targeting the cytokine signaling complex to help maximize efficacy and reduce side effects are required.

## 11. Conclusions

Th17 has demonstrated a significant contribution to the progression of autoimmune disease and inflammation in the gastrointestinal tract. IL-17, as the main cytokine from Th17 cell, has a pleiotropic effect in defensive and pathogenic gut homeostasis. In *H. pylori* infection, IL-17 is necessary for bacterial clearance; however, the dual nature of it function gives rise to inflammation-induced diseases. Together with IL-21 and IL-23, it plays a role in inflammation site formation, either directly or indirectly. From reviewing previous sources, this study identified interactions between cytokines and intestinal mucosa that require further understanding. Since it has an important role in gastroduodenal diseases, the comprehensive dynamic of IL-17 modulation needs to be considered carefully for the clinical outcome of gastritis and GC patients.

## Figures and Tables

**Table 1:** Source and known function of Th17-related cytokines.

Cytokine	Cell Producers	Played Roles	Mechanism of Action	
IL-17A, IL-17F	Th17	Mucosal protection	Increases bacterial elimination	[53,54]
			Induces claudin, mucin production	[14,63]
		Gastric inflammation	Recruitment of neutrophils	[74,82,83]
			Synergize with IFNγ & IL-8	[8,76,79]
		Progression of ulcer	Increases mucosal damages	[71]
		Progression of gastric cancer	Expression of angiogenic factors	[84]
			Promotes cancer cells’ growth	[85]
		Exacerbation of colitis	Increases chemokine	[36,70]
			Recruitment of neutrophil	[32,51]
IL-21	Th17, Th1, Tfh, NK,	Promotion to inflammation	Maintains Th17 and Th1 proliferation	[42]
			Induces CCL20	[70,86]
		Induction of *H. pylori*-specific antibodies	Autocrine loop of Tfh cell	[87]
			Drives B cell to produce antibodies	[88,89]
		ECM degradation	Induces MMPs	[11]
IL-23	Macrophage, DCs, neutrophil	Promotion to inflammation	Maintains Th17 and Th1 proliferation	[66,67,90]
			Recruitment of neutrophils	[14,82]

## Data Availability

Not applicable.

## References

[1] Correa P., Houghton J. (2007). Carcinogenesis of Helicobacter pylori. Gastroenterology.

[2] Suerbaum S., Smith J.M., Bapumia K., Morelli G., Smith N.H., Kunstmann E., Dyrek I., Achtman M. (1998). Free recombination within Helicobacter pylori. Proc. Natl. Acad. Sci. USA.

[3] Hatakeyama M., Higashi H. (2005). Helicobacter pylori CagA: A new paradigm for bacterial carcinogenesis. Cancer Sci..

[4] Watabe H., Mitsushima T., Yamaji Y., Okamoto M., Wada R., Kokubo T., Doi H., Yoshida H., Kawabe T., Omata M. (2005). Predicting the development of gastric cancer from combining Helicobacter pylori antibodies and serum pepsinogen status: A prospective endoscopic cohort study. Gut.

[5] Miftahussurur M., Waskito L.A., Syam A.F., Nusi I.A., Wibawa I.D.N., Rezkitha Y.A.A., Siregar G., Yulizal O.K., Akil F., Uwan W.B. (2019). Analysis of risks of gastric cancer by gastric mucosa among Indonesian ethnic groups. PLoS ONE.

[6] Savoldi A., Carrara E., Graham D.Y., Conti M., Tacconelli E. (2018). Prevalence of Antibiotic Resistance in Helicobacter pylori: A Systematic Review and Meta-analysis in World Health Organization Regions. Gastroenterology.

[7] Caruso R., Fina D., Paoluzi O.A., Blanco G.D.V., Stolfi C., Rizzo A., Caprioli F., Sarra M., Andrei F., Fantini M.C. (2008). IL-23-mediated regulation of IL-17 production inHelicobacter pylori-infected gastric mucosa. Eur. J. Immunol..

[8] Shi Y., Liu X.-F., Zhuang Y., Zhang J.-Y., Liu T., Yin Z., Wu C., Mao X.-H., Jia K.-R., Wang F.-J. (2010). Helicobacter pylori-Induced Th17 Responses Modulate Th1 Cell Responses, Benefit Bacterial Growth, and Contribute to Pathology in Mice. J. Immunol..

[9] Shabgah A.G., Fattahi E., Shahneh F.Z. (2014). Interleukin-17 in human inflammatory diseases. Adv. Dermatol. Allergol..

[10] Bagheri N., Azadegan-Dehkordi F., Shirzad H., Rafieian-Kopaei M., Rahimian G., Razavi A. (2015). The biological functions of IL-17 in different clinical expressions of Helicobacter pylori-infection. Microb. Pathog..

[11] Yi J.S., Cox M.A., Zajac A.J. (2010). Interleukin-21: A multifunctional regulator of immunity to infections. Microbes Infect..

[12] Hue S., Ahern P., Buonocore S., Kullberg M.C., Cua D.J., McKenzie B.S., Powrie F., Maloy K.J. (2006). Interleukin-23 drives innate and T cell–mediated intestinal inflammation. J. Exp. Med..

[13] Caruso R., Pallone F., Monteleone G. (2007). Emerging role of IL-23/IL-17 axis in H pylori-associated pathology. World J. Gastroenterol..

[14] Morrison P.J., Ballantyne S.J., Kullberg M.C. (2011). Interleukin-23 and T helper 17-type responses in intestinal inflammation: From cytokines to T-cell plasticity. Immunology.

[15] Koussoulas V., Vassiliou S., Giamarellos-Bourboulis E.J., Tassias G., Kotsaki A., Barbatzas C., Tzivras M. (2009). Implications for a role of interleukin-23 in the pathogenesis of chronic gastritis and of peptic ulcer disease. Clin. Exp. Immunol..

[16] Ümit H., Tezel A., Bukavaz S., Unsal G., Otkun M., Soylu A.R., Tucer D., Otkun M., Bilgi S. (2008). The Relationship Between Virulence Factors of Helicobacter pylori and Severity of Gastritis in Infected Patients. Dig. Dis. Sci..

[17] Chmiela M., Walczak N., Rudnicka K. (2018). Helicobacter pylori outer membrane vesicles involvement in the infection development and Helicobacter pylori-related diseases. J. Biomed. Sci..

[18] Wang S.-K., Zhu H.-F., He B.-S., Zhang Z.-Y., Chen Z.-T., Wang Z.-Z., Wu G.-L. (2007). CagA+ H pylori infection is associated with polarization of T helper cell immune responses in gastric carcinogenesis. World J. Gastroenterol..

[19] Brandt S., Kwok T., Hartig R., König W., Backert S. (2005). NF-κB activation and potentiation of proinflammatory responses by the *Helicobacter pylori* CagA protein. Proc. Natl. Acad. Sci. USA.

[20] Nilsson C., Sillén A., Eriksson L., Strand M.-L., Enroth H., Normark S., Falk P., Engstrand L. (2003). Correlation between cag Pathogenicity Island Composition and Helicobacter pylori-Associated Gastroduodenal Disease. Infect. Immun..

[21] Tanaka S., Nagashima H., Cruz M., Uchida T., Uotani T., Abreu J.A.J., Mahachai V., Vilaichone R.-K., Ratanachu-Ek T., Tshering L. (2017). Interleukin-17C in Human Helicobacter pylori Gastritis. Infect. Immun..

[22] Lina T.T., Pinchuk I.V., House J., Yamaoka Y., Graham D.Y., Beswick E.J., Reyes V.E. (2013). CagA-dependent downregulation of B7-H2 expression on gastric mucosa and inhibition of Th17 responses during Helicobacter pylori infection. J. Immunol..

[23] Caruso R., Fina D., Peluso I., Fantini M.C., Tosti C., Blanco G.D.V., Paoluzi O.A., Caprioli F., Andrei F., Stolfi C. (2007). IL-21 Is Highly Produced inHelicobacter pylori-Infected Gastric Mucosa and Promotes Gelatinases Synthesis. J. Immunol..

[24] Bagheri N., Azadegan-Dehkordi F., Shirzad M., Zamanzad B., Rahimian G., Taghikhani A., Rafieian-Kopaei M., Shirzad H. (2015). Clinical immunology Mucosal interleukin-21 mRNA expression level is high in patients with Helicobacter pylori and is associated with the severity of gastritis. Central Eur. J. Immunol..

[25] Kranzer K., Eckhardt A., Aigner M., Knoll G., Deml L., Speth C., Lehn N., Rehli M., Schneider-Brachert W. (2004). Induction of Maturation and Cytokine Release of Human Dendritic Cells by Helicobacter pylori. Infect. Immun..

[26] Andres S., Schmidt H.M., Mitchell H., Rhen M., Maeurer M., Engstrand L. (2011). Helicobacter pylori defines local immune response through interaction with dendritic cells. FEMS Immunol. Med. Microbiol..

[27] Kao J.Y., Zhang M., Miller M.J., Mills J.C., Wang B., Liu M., Eaton K.A., Zou W., Berndt B.E., Cole T.S. (2010). Helicobacter pylori Immune Escape Is Mediated by Dendritic Cell–Induced Treg Skewing and Th17 Suppression in Mice. Gastroenterology.

[28] Shiu J., Blanchard T.G. (2013). Dendritic cell function in the host response to Helicobacter pylori infection of the gastric mucosa. Pathog. Dis..

[29] Kim J.M., Kim J.S., Yoo D.Y., Ko S.H., Kim N., Kim H., Kim Y.-J. (2011). Stimulation of dendritic cells with Helicobacter pylori vacuolating cytotoxin negatively regulates their maturation via the restoration of E2F1. Clin. Exp. Immunol..

[30] Zhang J.-Y., Liu T., Guo H., Liu X.-F., Zhuang Y., Yu S., Chen L., Wu C., Zhao Z., Tang B. (2011). Induction of a Th17 cell response by Helicobacter pylori Urease subunit B. Immunobiology.

[31] Rong Z., Cheng L., Ren Y., Li Z., Li Y., Li X., Li H., Fu X.-Y., Chang Z. (2007). Interleukin-17F signaling requires ubiquitination of interleukin-17 receptor via TRAF6. Cell Signal..

[32] Gaffen S.L. (2009). Structure and signalling in the IL-17 receptor family. Nat. Rev. Immunol..

[33] Mangan P.R., Harrington L.E., O’Quinn D.B., Helms W.S., Bullard D.C., Elson C.O., Hatton R.D., Wahl S.M., Schoeb T.R., Weaver C.T. (2006). Transforming growth factor-beta induces development of the T(H)17 lineage. Nature.

[34] Volpe E., Servant N., Zollinger R., Bogiatzi S.I., Hupe P., Barillot E., Soumelis V. (2008). A critical function for transforming growth factor-beta, interleukin 23 and proinflammatory cytokines in driving and modulating human T(H)-17 responses. Nat. Immunol..

[35] Yao Z., Fanslow W.C., Seldin M.F., Rousseau A.-M., Painter S.L., Comeau M.R., Cohen J.I., Spriggs M.K. (2011). Herpesvirus saimiri encodes a new cytokine, IL-17, which binds to a novel cytokine receptor. J. Immunol..

[36] Awane M., Andres P.G., Li D.J., Reinecker H.C. (1999). NF-kappa B-inducing kinase is a common mediator of IL-17-, TNF-alpha-, and IL-1 beta-induced chemokine promoter activation in intestinal epithelial cells. J. Immunol..

[37] Shi J.H., Sun S.C. (2018). Tumor Necrosis Factor Receptor-Associated Factor Regulation of Nuclear Factor kappaB and Mitogen-Activated Protein Kinase Pathways. Front. Immunol..

[38] Schwandner R., Yamaguchi K., Cao Z. (2000). Requirement of Tumor Necrosis Factor Receptor–Associated Factor (Traf)6 in Interleukin 17 Signal Transduction. J. Exp. Med..

[39] Louten J., Boniface K., Malefyt R.D.W. (2009). Development and function of TH17 cells in health and disease. J. Allergy Clin. Immunol..

[40] Dixon B.R.E.A., Hossain R., Patel R.V., Algood H.M.S. (2019). Th17 Cells in Helicobacter pylori Infection: A Dichotomy of Help and Harm. Infect. Immun..

[41] Asao H., Okuyama C., Kumaki S., Ishii N., Tsuchiya S., Foster D., Sugamura K. (2001). Cutting edge: The common gamma-chain is an indispensable subunit of the IL-21 receptor complex. J. Immunol..

[42] Carbo A., Olivares-Villagómez D., Hontecillas R., Bassaganya-Riera J., Chaturvedi R., Piazuelo M.B., Delgado A., Washington M.K., Wilson K.T., Algood H.M.S. (2014). Systems Modeling of the Role of Interleukin-21 in the Maintenance of Effector CD4+ T Cell Responses during Chronic Helicobacter pylori Infection. mBio.

[43] Fina D., Sarra M., Fantini M.C., Rizzo A., Caruso R., Caprioli F., Stolfi C., Cardolini I., Dottori M., Boirivant M. (2008). Regulation of gut inflammation and th17 cell response by interleukin-21. Gastroenterology.

[44] Korn T., Bettelli E., Gao W., Awasthi A., Jager A., Strom T.B., Oukka M., Kuchroo V.K. (2007). IL-21 initiates an alternative pathway to induce proinflammatory T(H)17 cells. Nature.

[45] Noguchi M., Yi H., Rosenblatt H.M., Filipovich A.H., Adelstein S., Modi W.S., McBride O.W., Leonard W.J. (1993). Interleukin-2 receptor gamma chain mutation results in X-linked severe combined immunodeficiency in humans. Cell.

[46] Habib T., Senadheera S., Weinberg K., Kaushansky K. (2002). The common gamma chain (gamma c) is a required signaling component of the IL-21 receptor and supports IL-21-induced cell proliferation via JAK3. Biochemistry.

[47] Leonard W.J., Zeng R., Spolski R. (2008). Interleukin 21: A cytokine/cytokine receptor system that has come of age. J. Leukoc. Biol..

[48] Duvallet E., Semerano L., Assier E., Falgarone G., Boissier M.-C. (2011). Interleukin-23: A key cytokine in inflammatory diseases. Ann. Med..

[49] Parham C., Chirica M., Timans J., Vaisberg E., Travis M., Cheung J., Pflanz S., Zhang R., Singh K.P., Vega F. (2002). A receptor for the heterodimeric cytokine IL-23 is composed of IL-12Rbeta1 and a novel cytokine receptor subunit, IL-23R. J. Immunol..

[50] Ghoreschi K., Laurence A., Yang X.P., Tato C.M., McGeachy M.J., Konkel J.E., Ramos H.L., Wei L., Davidson T.S., Bouladoux N. (2010). Generation of pathogenic T(H)17 cells in the absence of TGF-beta signalling. Nature.

[51] Coccia M., Harrison O.J., Schiering C., Asquith M.J., Becher B., Powrie F., Maloy K.J. (2012). IL-1beta mediates chronic intestinal inflammation by promoting the accumulation of IL-17A secreting innate lymphoid cells and CD4(+) Th17 cells. J. Exp. Med..

[52] Cua D.J., Sherlock J.P., Chen Y., Murphy C.A., Joyce B., Seymour B.W.P., Lucian L., To W., Kwan S., Churakova T. (2003). Interleukin-23 rather than interleukin-12 is the critical cytokine for autoimmune inflammation of the brain. Nat. Cell Biol..

[53] Ishigame H., Kakuta S., Nagai T., Kadoki M., Nambu A., Komiyama Y., Fujikado N., Tanahashi Y., Akitsu A., Kotaki H. (2009). Differential Roles of Interleukin-17A and -17F in Host Defense against Mucoepithelial Bacterial Infection and Allergic Responses. Immunity.

[54] Solaymani-Mohammadi S., Berzofsky J.A. (2019). Interleukin 21 collaborates with interferon-gamma for the optimal expression of interferon-stimulated genes and enhances protection against enteric microbial infection. PLoS Pathog..

[55] Castaño-Rodríguez N., Kaakoush N.O., Lee W.S., Mitchell H.M. (2017). Dual role of Helicobacter and Campylobacter species in IBD: A systematic review and meta-analysis. Gut.

[56] Wu X.W., Ji H.Z., Yang M.F., Wu L., Wang F.Y. (2015). Helicobacter pylori infection and inflammatory bowel disease in Asians: A meta-analysis. World J. Gastroenterol..

[57] Yu Y., Zhu S., Li P., Min L., Zhang S. (2018). Helicobacter pylori infection and inflammatory bowel disease: A crosstalk between upper and lower digestive tract. Cell Death Dis..

[58] Rokkas T., Gisbert J.P., Niv Y., O’Morain C. (2015). The association between Helicobacter pylori infection and inflammatory bowel disease based on meta-analysis. United Eur. Gastroenterol. J..

[59] Ueno A., Jijon H., Chan R., Ford K., Hirota C., Kaplan G.G., Beck P.L., Iacucci M., Gasia M.F., Barkema H.W. (2013). Increased Prevalence of Circulating Novel IL-17 Secreting Foxp3 Expressing CD4+ T Cells and Defective Suppressive Function of Circulating Foxp3+ Regulatory Cells Support Plasticity Between Th17 and Regulatory T Cells in Inflammatory Bowel Disease Patients. Inflamm. Bowel Dis..

[60] Jiang W., Su J., Zhang X., Cheng X., Zhou J., Shi R., Zhang H. (2014). Elevated levels of Th17 cells and Th17-related cytokines are associated with disease activity in patients with inflammatory bowel disease. Inflamm. Res..

[61] Gil J.H., Seo J.W., Cho M.-S., Ahn J.-H., Sung H.Y. (2014). Role of Treg and TH17 Cells of the Gastric Mucosa in Children with Helicobacter pylori Gastritis. J. Pediatr. Gastroenterol. Nutr..

[62] Zhang H., Dai Y., Liu M.Y., Wu T., Li J., Wang X., Wang W. (2018). Helicobacter pylori Colonization Protects Against Chronic Experimental Colitis by Regulating Th17/Treg Balance. Inflamm. Bowel Dis..

[63] Dixon B.R.E.A., Radin J.N., Piazuelo M.B., Contreras D.C., Algood H.M.S. (2016). IL-17a and IL-22 Induce Expression of Antimicrobials in Gastrointestinal Epithelial Cells and May Contribute to Epithelial Cell Defense against Helicobacter pylori. PLoS ONE.

[64] Abraham C., Cho J. (2009). Interleukin-23/Th17 pathways and inflammatory bowel disease. Inflamm. Bowel Dis..

[65] Bank S., Andersen P.S., Burisch J., Pedersen N., Roug S., Galsgaard J., Turino S.Y., Brodersen J.B., Rashid S., Rasmussen B.K. (2015). Polymorphisms in the Toll-Like Receptor and the IL-23/IL-17 Pathways Were Associated with Susceptibility to Inflammatory Bowel Disease in a Danish Cohort. PLoS ONE.

[66] Lee J.S., Tato C.M., Joyce-Shaikh B., Gulen M.F., Cayatte C., Chen Y., Blumenschein W.M., Judo M., Ayanoglu G., McClanahan T.K. (2015). Interleukin-23-Independent IL-17 Production Regulates Intestinal Epithelial Permeability. Immunity.

[67] Stritesky G.L., Yeh N., Kaplan M.H. (2008). IL-23 promotes maintenance but not commitment to the Th17 lineage 1. J. Immunol..

[68] Ahern P.P., Schiering C., Buonocore S., McGeachy M.J., Cua D.J., Maloy K.J., Powrie F. (2010). Interleukin-23 Drives Intestinal Inflammation through Direct Activity on T Cells. Immunity.

[69] Monteleone G., Pallone F., Macdonald T.T. (2009). Interleukin-21 (IL-21)-mediated pathways in T cell-mediated disease. Cytokine Growth Factor Rev..

[70] Hirota K., Yoshitomi H., Hashimoto M., Maeda S., Teradaira S., Sugimoto N., Yamaguchi T., Nomura T., Ito H., Nakamura T. (2007). Preferential recruitment of CCR6-expressing Th17 cells to inflamed joints via CCL20 in rheumatoid arthritis and its animal model. J. Exp. Med..

[71] Liu C., Zhang Z., Zhu M. (2016). Immune Responses Mediated by Th17 Cells in *Helicobacter pylori* Infection. Integr. Med. Int..

[72] Iwakura Y., Nakae S., Saijo S., Ishigame H. (2008). The roles of IL-17A in inflammatory immune responses and host defense against pathogens. Immunol. Rev..

[73] Zhou L., Lopes J.E., Chong M.M.W., Ivanov I.I., Min R., Victora G.D., Shen Y., Du J., Rubtsov Y.P., Rudensky A.Y. (2008). TGF-β-induced Foxp3 inhibits TH17 cell differentiation by antagonizing RORγt function. Nat. Cell Biol..

[74] DeLyria E.S., Redline R.W., Blanchard T.G. (2009). Vaccination of Mice Against H pylori Induces a Strong Th-17 Response and Immunity That Is Neutrophil Dependent. Gastroenterology.

[75] Bagheri N., Shirzad H., Elahi S., Azadegan-Dehkordi F., Rahimian G., Shafigh M., Rashidii R., Sarafnejad A., Rafieian-Kopaei M., Faridani R. (2017). Downregulated regulatory T cell function is associated with increased peptic ulcer in Helicobacter pylori-infection. Microb. Pathog..

[76] Sebkova L., Pellicanò A., Monteleone G., Grazioli B., Guarnieri G., Imeneo M., Pallone F., Luzza F. (2004). Extracellular Signal-Regulated Protein Kinase Mediates Interleukin 17 (IL-17)-Induced IL-8 Secretion in Helicobacter pylori-Infected Human Gastric Epithelial Cells. Infect. Immun..

[77] Siddique I., Al-Qabandi A., Al-Ali J., Alazmi W., Memon A., Mustafa A.S., Junaid T.A. (2014). Association between Helicobacter pylori genotypes and severity of chronic gastritis, peptic ulcer disease and gastric mucosal interleukin-8 levels: Evidence from a study in the Middle East. Gut Pathog..

[78] Bagheri N., Razavi A., Pourgheysari B., Azadegan-Dehkordi F., Rahimian G., Pirayesh A., Shafigh M., Rafieian-Kopaei M., Fereidani R., Tahmasbi K. (2018). Up-regulated Th17 cell function is associated with increased peptic ulcer disease in Helicobacter pylori -infection. Infect. Genet. Evol..

[79] Andoh A., Takaya H., Makino J., Sato H., Bamba S., Araki Y., Hata K., Shimada M., Okuno T., Fujiyama Y. (2001). Cooperation of interleukin-17 and interferon-gamma on chemokine secretion in human fetal intestinal epithelial cells. Clin. Exp. Immunol..

[80] Lee K.E., Khoi P.N., Xia Y., Park J.S., Joo Y.E., Kim K.K., Choi S.Y., Jung Y.D. (2013). Helicobacter pylori and interleukin-8 in gastric cancer. World J. Gastroenterol..

[81] Butcher L.D., Hartog G.D., Ernst P.B., Crowe S.E. (2017). Oxidative Stress Resulting From Helicobacter pylori Infection Contributes to Gastric Carcinogenesis. Cell. Mol. Gastroenterol. Hepatol..

[82] Iwakura Y., Ishigame H. (2006). The IL-23/IL-17 axis in inflammation. J. Clin. Investig..

[83] Li S., Cong X., Gao H., Lan X., Li Z., Wang W., Song S., Wang Y., Li C., Zhang H. (2019). Tumor-associated neutrophils induce EMT by IL-17a to promote migration and invasion in gastric cancer cells. J. Exp. Clin. Cancer Res..

[84] Rezalotfi A., Ahmadian E., Aazami H., Solgi G., Ebrahimi M. (2019). Gastric Cancer Stem Cells Effect on Th17/Treg Balance; A Bench to Beside Perspective. Front. Oncol..

[85] Wang Y., Wu H., Wu X., Bian Z., Gao Q. (2014). Interleukin 17A promotes gastric cancer invasiveness via NF-kappaB mediated matrix metalloproteinases 2 and 9 expression. PLoS ONE.

[86] Cook K.W., Letley D.P., Ingram R.J.M., Staples E., Skjoldmose H., Atherton J.C., Robinson K. (2014). CCL20/CCR6-mediated migration of regulatory T cells to theHelicobacter pylori-infected human gastric mucosa. Gut.

[87] Vogelzang A., McGuire H.M., Yu D., Sprent J., Mackay C.R., King C. (2008). A Fundamental Role for Interleukin-21 in the Generation of T Follicular Helper Cells. Immunity.

[88] Yasmin S., Dixon B.R.E.A., Olivares-Villagómez D., Algood H.M.S. (2019). Interleukin-21 (IL-21) Downregulates Dendritic Cell Cytokine Responses to Helicobacter pylori and Modulates T Lymphocyte IL-17A Expression in Peyer’s Patches during Infection. Infect. Immun..

[89] Algood H.M.S., Allen S.S., Washington M.K., Peek R.M., Miller G.G., Cover T.L. (2009). Regulation of Gastric B Cell Recruitment Is Dependent on IL-17 Receptor A Signaling in a Model of Chronic Bacterial Infection. J. Immunol..

[90] Gaffen S.L., Jain R., Garg A.V., Cua D.J. (2014). The IL-23–IL-17 immune axis: From mechanisms to therapeutic testing. Nat. Rev. Immunol..

[91] Pinchuk I.V., Morris K.T., Nofchissey R.A., Earley R.B., Wu J.-Y., Ma T.Y., Beswick E.J. (2013). Stromal Cells Induce Th17 during Helicobacter pylori Infection and in the Gastric Tumor Microenvironment. PLoS ONE.

[92] Wang J., Blanchard T.G., Ernst P.B., Mobley H.L.T., Mendz G.L., Hazell S.L. (2001). Host Inflammatory Response to Infection. Helicobacter Pylori: Physiology and Genetics.

[93] Negrini R., Lisato L., Zanella I., Cavazzini L., Gullini S., Villanacci V., Poiesi C., Albertini A., Ghielmi S. (1991). Helicobacter pylori infection induces antibodies cross-reacting with human gastric mucosa. Gastroenterology.

[94] Akhiani A.A., Schön K., Franzén L.E., Pappo J., Lycke N. (2004). Helicobacter pylori-Specific Antibodies Impair the Development of Gastritis, Facilitate Bacterial Colonization, and Counteract Resistance against Infection. J. Immunol..

[95] Pontarini E., Murray-Brown W.J., Croia C., Lucchesi D., Conway J., Rivellese F., Fossati-Jimack L., Astorri E., Prediletto E., Corsiero E. (2020). Unique expansion of IL-21+ Tfh and Tph cells under control of ICOS identifies Sjögren’s syndrome with ectopic germinal centres and MALT lymphoma. Ann. Rheum. Dis..

[96] Khamri W., Walker M.M., Clark P., Atherton J.C., Thursz M.R., Bamford K.B., Lechler R.I., Lombardi G. (2009). Helicobacter pylori Stimulates Dendritic Cells To Induce Interleukin-17 Expression from CD4+ T Lymphocytes. Infect. Immun..

[97] Horvath D.J.J., Washington M.K., Cope V.A., Algood H.M.S. (2012). IL-23 Contributes to Control of Chronic Helicobacter Pylori Infection and the Development of T Helper Responses in a Mouse Model1. Front. Immunol..

[98] Cho M.L., Kang J.W., Moon Y.M., Nam H.J., Jhun J.Y., Heo S.B., Jin H.T., Min S.Y., Ju J.H., Park K.S. (2006). STAT3 and NF-kappaB signal pathway is required for IL-23-mediated IL-17 production in spontaneous arthritis animal model IL-1 receptor antagonist-deficient mice. J. Immunol..

[99] Zhou L., Ivanov I.I., Spolski R., Min R., Shenderov K., Egawa T., Levy D.E., Leonard W.J., Littman D.R. (2007). IL-6 programs T(H)-17 cell differentiation by promoting sequential engagement of the IL-21 and IL-23 pathways. Nat. Immunol..

[100] Uemura N., Okamoto S., Yamamoto S., Matsumura N., Yamaguchi S., Yamakido M., Taniyama K., Sasaki N., Schlemper R.J. (2001). Helicobacter pyloriInfection and the Development of Gastric Cancer. N. Engl. J. Med..

[101] Maruyama T., Kono K., Mizukami Y., Kawaguchi Y., Mimura K., Watanabe M., Izawa S., Fujii H. (2010). Distribution of Th17 cells and FoxP3(+) regulatory T cells in tumor-infiltrating lymphocytes, tumor-draining lymph nodes and peripheral blood lymphocytes in patients with gastric cancer. Cancer Sci..

[102] Li Q., Li Q., Chen J., Liu Y., Zhao X., Tan B., Ai J., Zhang Z., Song J., Shan B. (2013). Prevalence of Th17 and Treg cells in gastric cancer patients and its correlation with clinical parameters. Oncol. Rep..

[103] Zhang B., Rong G., Wei H., Zhang M., Bi J., Ma L., Xue X., Wei G., Liu X., Fang G. (2008). The prevalence of Th17 cells in patients with gastric cancer. Biochem. Biophys. Res. Commun..

[104] Dai Z.M., Zhang T.S., Lin S., Zhang W.G., Liu J., Cao X.M., Li H.B., Wang M., Liu X.H., Liu K. (2016). Role of IL-17A rs2275913 and IL-17F rs763780 polymorphisms in risk of cancer development: An updated meta-analysis. Sci. Rep..

[105] Amedei A., Munari F., Della Bella C., Niccolai E., Benagiano M., Bencini L., Cianchi F., Farsi M., Emmi G., Zanotti G. (2012). Helicobacter pylori secreted peptidyl prolyl cis, trans-isomerase drives Th17 inflammation in gastric adenocarcinoma. Intern. Emerg. Med..

[106] Yan L., Zhang J., Guo D., Ma J., Shui S.-F., Han X.-W. (2018). IL-21R functions as an oncogenic factor and is regulated by the lncRNA MALAT1/miR-125a-3p axis in gastric cancer. Int. J. Oncol..

[107] Błogowski W., Madej-Michniewicz A., Marczuk N., Dołęgowska B., Starzyńska T. (2016). Interleukins 17 and 23 in patients with gastric neoplasms. Sci. Rep..

[108] Liu C., Zhang Y., Zhan J., Zhao Y., Wan Q., Peng H., Zhu W. (2014). Interleukin-23A is associated with tumor growth in Helicobacter-pylori-related human gastric cancer. Cancer Cell Int..

[109] Xu X., Yang C., Chen J., Liu J., Li P., Shi Y., Yu P. (2018). Interleukin-23 promotes the migration and invasion of gastric cancer cells by inducing epithelial-to-mesenchymal transition via the STAT3 pathway. Biochem. Biophys. Res. Commun..

[110] Mommersteeg M.C., Yu J., Peppelenbosch M.P., Fuhler G.M. (2018). Genetic host factors in Helicobacter pylori -induced carcinogenesis: Emerging new paradigms. Biochim. Biophys. Acta (BBA) Bioenerg..

[111] Zhou F., Qiu L.-X., Cheng L., Wang M.-Y., Li J., Sun M.-H., Yang Y.-J., Wang J.-C., Jin L., Wang Y.-N. (2016). Associations of genotypes and haplotypes of IL-17 with risk of gastric cancer in an eastern Chinese population. Oncotarget.

[112] Elshazli R.M., Salman D.O., Kamel M.M., Toraih E.A., Fawzy M.S. (2018). Genetic polymorphisms of IL-17A rs2275913, rs3748067 and IL-17F rs763780 in gastric cancer risk: Evidence from 8124 cases and 9873 controls. Mol. Biol. Rep..

[113] Feng B., Fan Y., Wang W., Yao G., Zhai J. (2014). IL-17A G197A and C1249T polymorphisms in gastric carcinogenesis. Tumor Biol..

[114] Gao J.-F., Zhang H., Lv J., Wang L., Fan Y.-Y. (2019). Associations of the IL-17A rs2275913 and IL-17F rs763780 polymorphisms with the risk of digestive system neoplasms: A meta-analysis. Int. Immunopharmacol..

[115] Wang X.-Q., Li Y., Terry P.D., Kou W.-J., Zhang Y., Hui Z.-Z., Ren X.-H., Wang M.-X. (2020). Association between interleukin-21 gene rs907715 polymorphism and gastric precancerous lesions in a Chinese population. World J. Gastrointest. Oncol..

[116] He B., Pan B., Pan Y., Wang X., Zhou L., Sun H., Xu T., Xu X., Liu X., Wang S. (2019). Polymorphisms of IL-23R predict survival of gastric cancer patients in a Chinese population. Cytokine.

[117] Robinson K., Kenefeck R., Pidgeon E.L., Shakib S., Patel S., Polson R.J., Zaitoun A.M., Atherton J.C. (2008). Helicobacter pylori-induced peptic ulcer disease is associated with inadequate regulatory T cell responses. Gut.

[118] Rad R., Brenner L., Bauer S., Schwendy S., Layland L., da Costa C.P., Reindl W., Dossumbekova A., Friedrich M., Saur D. (2006). CD25+/Foxp3+ T Cells Regulate Gastric Inflammation and Helicobacter pylori Colonization In Vivo. Gastroenterology.

[119] Nakano H., Hirata I., Okubo M., Arima Y., Kamiya Y., Fujita H., Yoshioka D., Nakamura M., Nagasaka M., Shibata T. (2007). Genetic polymorphisms of molecules associated with inflammation and immune response in Japanese subjects with functional dyspepsia. Int. J. Mol. Med..

[120] Meliț L.E., Mărginean C.O., Mărginean C.D., Mărginean M.O. (2019). The Relationship between Toll-like Receptors and Helicobacter pylori-Related Gastropathies: Still a Controversial Topic. J. Immunol. Res..

[121] Fauny M., Moulin D., D’Amico F., Netter P., Petitpain N., Arnone D., Jouzeau J.-Y., Loeuille D., Peyrin-Biroulet L. (2020). Paradoxical gastrointestinal effects of interleukin-17 blockers. Ann. Rheum. Dis..

[122] Allocca M., Furfaro F., Fiorino G., Gilardi D., D’Alessio S., Danese S. (2018). Can IL-23 be a good target for ulcerative colitis?. Best Pract. Res. Clin. Gastroenterol..

[123] Bockerstett K.A., Osaki L.H., Petersen C.P., Cai C.W., Wong C.F., Nguyen T.-L.M., Ford E.L., Hoft D.F., Mills J.C., Goldenring J.R. (2018). Interleukin-17A Promotes Parietal Cell Atrophy by Inducing Apoptosis. Cell. Mol. Gastroenterol. Hepatol..

